# Is the population of Sado Island genetically close to the population of western Japan?

**DOI:** 10.1038/s41439-019-0058-6

**Published:** 2019-06-04

**Authors:** Kazuharu Misawa, Hiroshi Watanabe, Akio Yokoseki, Minako Wakasugi, Osamu Onodera, Ichiei Narita, Takeshi Momotsu, Kenji Sato, Naoto Endo

**Affiliations:** 1 Department of Integrative Genomics, Tohoku Medical Megabank Organization, Tohoku University, Sendai, Miyagi Japan; 2 Department of Cardiovascular Biology and Medicine, Niigata University Graduate School of Medical and Dental Sciences, Niigata, Japan; 3 Department of Inter-Organ Communication Research, Niigata University Graduate School of Medical and Dental Sciences, Niigata, Japan; 4 Division of Comprehensive Geriatrics in Community, Niigata University Graduate School of Medical and Dental Sciences, Niigata, Japan; 5 Department of Neurology, Brain Research Institute, Niigata University, Niigata, Japan; 6 Division of Clinical Nephrology and Rheumatology, Niigata University Graduate School of Medical and Dental Sciences, Niigata, Japan; 7 Sado General Hospital, Niigata, Japan; 8 Division of Orthopedic Surgery, Department of Regenerative and Transplant Medicine, Niigata University Graduate School of Medical and Dental Sciences, Niigata, Japan; 9 Present Address: Kansai Medical University, 2-5-1 Shin-machi, Hirakata, 573-1010 Osaka Japan

**Keywords:** Genetic variation, Evolutionary biology

## Abstract

To explore the effect of aging, a cohort study is being performed on Sado Island, which is located in the Sea of Japan. Sado Island is close to the eastern coast of Japan, yet its population speaks the western Japanese dialect. Consequently, the genetic background of the population of Sado Island is of interest. Based on Nei’s genetic distance, we compared the allele frequencies of people from Sado Island to those of people from Nagahama and Miyagi, which are located in the western and northeastern parts of Honshu, respectively. The results showed that the populations of Miyagi and Nagahama are genetically closer to each other than to the population of Sado Island. Because the Sado and Honshu Islands are isolated by a channel, it is possible that genetic drift occurred within Sado Island, which would explain the uniqueness of the people of this region.

Japan is experiencing a “super-aging” society^1^. To explore the effect of aging, a cross-sectional study was performed, which was a subanalysis of the Project in Sado for Total Health (PROST), a hospital-based cohort study in Sado City, Niigata Prefecture, Japan (http://square.umin.ac.jp/prost/). PROST, a cohort study targeting outpatients (age ≥20 years) of Sado General Hospital^2^, began in June 2008 and is currently ongoing.

Sado Island is located 30 km off the coast of Niigata City, which is on the eastern coast of Honshu, mainland Japan. Despite their proximity, the dialect spoken on Sado Island is different from that spoken in Niigata^3^. Figure 1 shows the location of Sado Island and the distribution of Japanese dialects summarized in a previous study^4^. We hypothesized that the cultural difference reflects a difference in the ancestors of these populations. In this study, we investigated the genetic distances between the population of Sado Island and populations in eastern and western Japan.

**Fig. 1:**
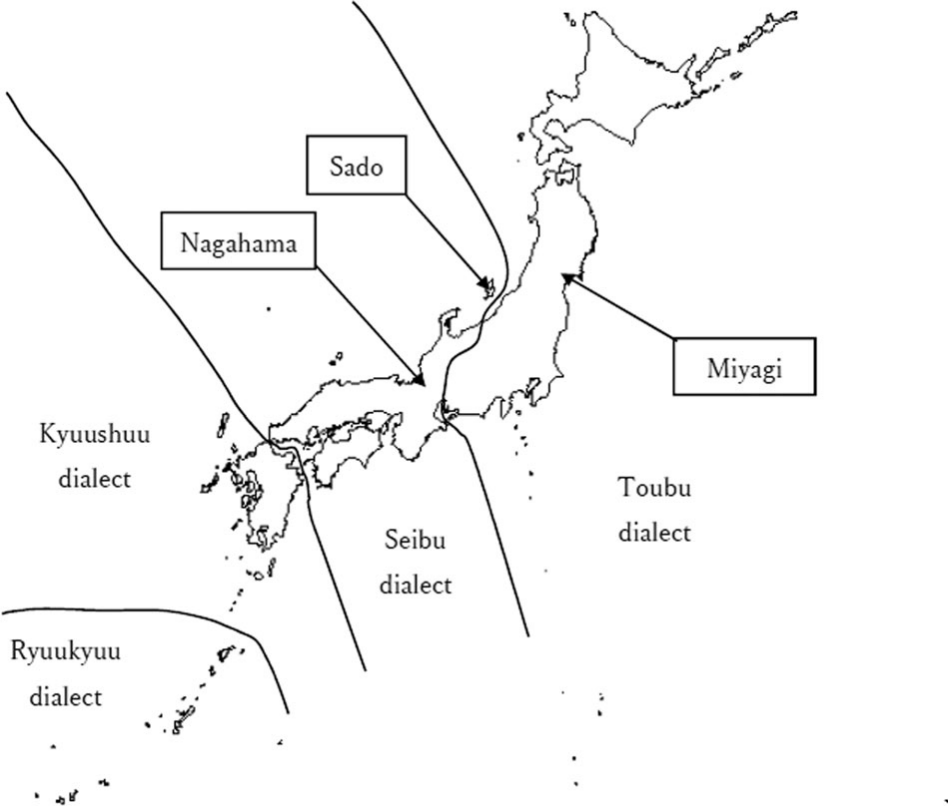
Locations of Sado Island, Miyagi, and Nagahama Dialect groups (Toubu, Seibu, Kyuushuu, and Ryuukyuu) summarized in the literature.^4^ The map was made with Natural Earth (naturalearthdata.com)

This project was part of the PROST study, and it received the approval of the ethics committee of Niigata University School of Medicine (No. G2015-0811) as well as the ethics committee of Tohoku Medical Megabank Project (No. 2018-4-78). The samples used were obtained from the cohort participants, who gave their written consent. In addition, genotype data were securely controlled under the Materials and Information Distribution Review Committee of Tohoku Medical Megabank Project, and data sharing with researchers was discussed with the review committee. (https://www.megabank.tohoku.ac.jp/wp/wp-content/uploads/2018/05/2017-1014.pdf).

We used the Japonica SNP array^5^ to genotype the participants in PROST according to the manufacturer’s instructions. In the present study, 1750 individuals were genotyped. All genotyped samples passed the recommended sample quality control metric for the AXIOM arrays (dish QC40.82); we excluded three control samples with an overall call rate of <99%. We recalled the remaining 1747 samples with Genotyping Console 4.2.0.26 software (Affymetrix). We performed LD pruning using PLINK 2.00 alpha with the option “–indep-pairwise 50 10 0.1.” We also excluded loci when their minor allele frequencies (MAF) were lower than 5% and when the loci failed (*P* < 0.05) the Hardy–Weinberg test. In total, 5,078 loci passed these filters.

As a representative of the western Japan population, allele frequencies were obtained from the Human Genetic Variation Database^6^, which contains the genotype counts of participants in The Nagahama Prospective Genome Cohort for the Comprehensive Human Bioscience^7^. In the present study, this dataset is referred to as Nagahama. As a representative of the eastern Japan population, we used iJGVD^8^, which contains data obtained from the Miyagi prefecture^9^. This dataset is referred to as Miyagi in this study. The locations of Miyagi and Nagahama are shown in Fig. 1. Note that both Miyagi and Nagahama are located in Honshu.

Nei’s genetic distance^10^ was calculated among the populations using all loci, and 99% confidence intervals of the Dst were calculated by bootstrap resampling, with 5,000 replications.

Table 1 shows the genetic distances and their confidence intervals (99%) among the populations of Sado, Miyagi, and Nagahama. The results showed that the genetic distances between the population on Sado Island and the Honshu populations were significantly larger than that between the Miyagi and Nagahama populations.

**Table 1:** Genetic distances and their confidence intervals (CI) among the Sado, Miyagi, and Nagahama populations

	Distance	99.9% CI
Sado–Miyagi	0.000331	0.000304–0.000358
Sado–Nagahama	0.000358	0.000327–0.000392
Miyagi–Nagahama	0.000167	0.000146–0.000190

In addition, Table 1 shows that the genetic distance between the Sado and Nagahama populations was not significantly different from that between the Sado and Miyagi populations. Thus, the hypothesis that the Sado population is genetically closer to the population in western Japan than the population in eastern Japan was neither rejected nor supported. We confirmed that the participants in PROST on Sado Island were genetically closer to Japanese living in Tokyo (JPT) than to Han Chinese in Beijing (CHB) and Southern Chinese (CHS) using principal component analysis (PCA) (Supplementary Fig. 1).

To estimate the degree of isolation of the Sado population, we estimated the inbreeding coefficients (IBC) based on the SNP homozygosity of samples using plink version 1.90b6.9. The same set of SNPs originally selected for PCA was used (Supplementary Fig. 1). The means ± SDs of the estimated IBC of the Sado and JPT populations are −0.0041 ± 0.0656 and −0.0043 ± 0.0129, respectively. The difference in IBC between the two populations was not statistically significant (*t*-test, *P* > 0.05). In other words, the size of the population on Sado Island is not overly small.

This study shows that geographic distance and linguistic similarity do not reflect the genetic differences among these populations. In addition, genetic drift may be more substantial than the linguistic shift.

## Supplementary information

Supplementary Fig. 1
