# Supplementary material for: Is the population of Sado Island genetically close to the population of western Japan?
Source: Hum Genome Var. 2019 Jun 4;6:26. doi: 10.1038/s41439-019-0058-6 (PMC6547765; doi:10.1038/s41439-019-0058-6)
Supplement: Supplementary file 1 — Supplementary Fig. 1 [file 41439_2019_58_MOESM1_ESM.docx]

Supplementary Fig. 1. Population structure analysis of the samples of reference panel and imputation subject

PCA analysis was performed using the plink 2.00 alpha in 1,747 people from Sado Island together with the samples of five populations of the International 1000 Genomes Project (1000G), namely, Japanese in Tokyo (JPT; n = 104), Han Chinese in Beijing (CHB; n = 103), Southern Han Chinese (CHS; n = 105), Yoruba in Ibadan, Nigeria (YRI, n = 108), and Utah Residents with Northern and Western European Ancestry (CEU, n = 99). The whole genome sequences of phase 3 of 1000G were retrieved from the FTP site. We filtered the SNPs with a minor allele frequency of >5%, genotype success rate of >99%, and Hardy-Weinberg equilibrium test P-value of >0.001 from the combined data. Finally, we obtained 329,791 SNPs after LD-pruning using plink 2.00 alpha program with "--indep-pairwise 50 10 0.01" option. Upper panel shows the first two components from principal component analysis (PCA) of the study samples. The lower panel is enlarged view of the areas around the East Asian populations (Sado, JPT, CHB, and CHS). The PCA plot shows that the Sado population is in the same cluster with JPT.
